# Baseline evidence practice gap for type 2 diabetes care among Aboriginal Australians in a cluster randomised controlled trial

**DOI:** 10.1186/1472-6963-14-S2-P33

**Published:** 2014-07-07

**Authors:** Sandra Eades, Chris Paul, Paul Ishiguchi, Paul Zimmet, Jonathan Shaw, Kristy Forshaw, Claudia Koller, Heidi Turon, Robert Sanson-Fisher

**Affiliations:** 1 Baker IDI Heart and Diabetes Institute, Melbourne, Victoria, Australia; 2 University of Newcastle, Newcastle, New South Wales, Australia

## Background

Type 2 diabetes is a major health problem in the Australian Indigenous population. Aboriginal Community Controlled Health Organisations (ACCHOs) are a primary care setting where there is opportunity to partner with health services to reduce the current evidence-practice gap in the provision of health care for Type 2 diabetes. The aim is to examine the effectiveness of a tailored ecologically-based collaborative model in achieving adherence to best practice clinical guidelines for Type 2 diabetes in ACCHOs. This study will examine whether the model results in improvements in diagnostic testing, monitoring and control of diabetes using reliable objective clinical indicators.

## Materials and methods

ACCHOs across Australia who use the Communicare data management system, have at least one doctor providing health care and use an electronic system for pathology results will be eligible for the study. A cluster randomised wait -control design will be used in 18 ACCHOs (9 intervention and 9 wait control). Cross-sectional measurement of the proportion of eligible patients receiving National Health and Medical Research Council (NHMRC) recommended diabetes diagnostic testing, monitoring and control at each ACCHO will be completed at baseline and follow-up.

## Results

At baseline the mean proportion of patients from clinics who: 1) received diagnostic testing was 58.5% (SD 23.4%) in intervention clinics and 71.4% (SD 14.5%) in control clinics; 2) appropriate monitoring for type 2 diabetes was 49.1% (SD 12.4%) in intervention clinics and 50.7% (SD 18.4%) in control clinics; and 3) appropriate control was 29.2% (SD 7.1 %) in intervention clinics and 34.2% (SD 8.4%) in control clinics.

## Conclusion

A significant evidence practice gap exists in this setting with a vulnerable population for type 2 diabetes care. The study has a number of potentially significant outcomes including the provision of a model for engaging ACCHOs in examining performance and improving their implementation of best evidence-practice; the development of resources such as staff orientation manuals; the use of naturally occurring reliable data sets for monitoring and feedback and as a tool for intervention delivery and outcome evaluation which may be generalisable to other populations.

